# PapMV nanoparticles improve mucosal immune responses to the trivalent inactivated flu vaccine

**DOI:** 10.1186/1477-3155-12-19

**Published:** 2014-05-03

**Authors:** Gervais Rioux, Claudia Mathieu, Alexis Russell, Marilène Bolduc, Marie-Eve Laliberté-Gagné, Pierre Savard, Denis Leclerc

**Affiliations:** 1 Department of Microbiology, Infectiology and Immunology, ‘Centre de recherche en Infectiologie’, Laval University, 2705 boul. Laurier, Quebec City, PQ G1V 4G2, Canada; 2 Neurosciences, Laval University, Quebec City, PQ, Canada

**Keywords:** PapMV nanoparticles, Adjuvant, Mucosal vaccine, Influenza, Trivalent inactivated flu vaccine, TIV, Seasonal flu vaccine, Mucosal immunity

## Abstract

**Background:**

Trivalent inactivated flu vaccines (TIV) are currently the best means to prevent influenza infections. However, the protection provided by TIV is partial (about 50%) and it is needed to improve the efficacy of protection. Since the respiratory tract is the main site of influenza replications, a vaccine that triggers mucosal immunity in this region can potentially improve protection against this disease. Recently, PapMV nanoparticles used as an adjuvant in a formulation with TIV administered by the subcutaneous route have shown improving the immune response directed to the TIV and protection against an influenza challenge.

**Findings:**

In the present study, we showed that intranasal instillation with a formulation containing TIV and PapMV nanoparticles significantly increase the amount of IgG, IgG2a and IgA in lungs of vaccinated mice as compared to mice that received TIV only. Instillation with the adjuvanted formulation leads to a more robust protection against an influenza infection with a strain that is lethal to mice vaccinated with the TIV.

**Conclusions:**

We demonstrate for the first time that PapMV nanoparticles are an effective and potent mucosal adjuvant for vaccination.

## Background

Vaccination with trivalent inactivated flu vaccines (TIV) remains the most affordable and efficient way to control diseases caused by influenza virus. The TIV is reformulated on the recommendation of WHO at the beginning of every year based on the circulating strains of the virus in the population. From that point, the manufacture process takes up to 8 months before the release the vaccine [1]. During this period, strains change through antigenic drift and shift and consequently, contribute to the decrease of vaccine effectiveness.

The TIV are currently administered by the intramuscular route, which is not optimal for protection to an influenza infection that occurs in the respiratory tract. The favored route of vaccination to trigger mucosal immunity in the respiratory tract is the intranasal route [2,3]. However, the TIV was previously showed to be less immunogenic by the intranasal route [4,5]. The amount of antigen needed to trigger IgA production by this route must be increased by two fold which, directly impact the number of available doses [4,5]. Therefore, changes in the vaccine formulation to improve TIV efficacy in the respiratory tracts and increase the number of doses are needed [6].

Development of mucosal adjuvants that can improve both, the immune response to TIV in lungs and dose sparing became a priority [5,7,8]. Current available adjuvants are ineffective in inducing mucosal immunity because they cannot be administered by the intranasal route due to their biophysical properties. As an example, aluminium hydroxide, the most commonly used adjuvant in vaccines, is inefficient with TIV and cannot be used by the mucosal route [9]. New safe and potent adjuvants are therefore needed.

In this study, we evaluated the potential of papaya mosaic virus (PapMV) nanoparticles to be used as a mucosal adjuvant. This hypothesis is supported by a previous report by our group showing that PapMV nanoparticles can trigger innate immunity in lungs of mice [10]. Instillation with PapMV nanoparticles triggered pro-inflammatory cytokines and chemokines secretion and immune cells recruitment shortly after treatment [10]. PapMV nanoparticles were also showed to be a TLR7 ligand, a receptor that activates innate immunity [11]. Furthermore, PapMV nanoparticles administered by the subcutaneous route were previously shown to improve and broaden the immune response to TIV in mouse and ferret animal models [7].

The objective of this study was to demonstrate that PapMV nanoparticles are able to induce mucosal immunity against TIV when administered by the intranasal route, in order to show its potential as a mucosal adjuvant. This route of administration was compared with subcutaneous injections as previously reported by our group [7].

## Methods

### PapMV nanoparticles production

PapMV nanoparticles were kindly provided by Folia Biotech Inc. (lot # L-5728, Quebec City, Canada). Those PapMV nanoparticles are comparable to a lot used in previous studies [10]. In brief, PapMV nanoparticles are constituted of PapMV coat proteins and assemble *in vitro* around an RNA transcript. The *Limulus Amebocyte* lysate (LAL) assay (Lonza, Walkersville, Maryland, USA) was used to evaluate the levels of LPS contaminants that were found to be below the detection limit. PapMV nanoparticles were characterized by electron microscopy and dynamic light scattering as described previously [12]. In brief, nanoparticles sizes were recorded with a ZetaSizer Nano ZS (Malvern, Worcestershire, United Kingdom) at 0.1 mg/ml in 10 mM Tris–HCl pH 8.0. Water diluted PapMV nanoparticles were stained with 3% acetate-uranyl on carbon-formvar grids then observed with a FEI-TECNAI-Spirit transmission electron microscope (FEI, Hillsboro, Oregon, USA).

### Vaccination protocol

Mice, 6 to 8-week-old BALB/c (10/group), were immunized twice at 14-day interval by the intranasal (i.n.) or subcutaneous (s.c.) routes with 50 μl of a formulation containing 2.1 μg of each hemagglutinin (HA) (TIV 2009–2010 (A/Brisbane/59/2007 (H1N1), A/Brisbane/10/2007 (H3N2), B/Brisbane/60/2008), cat #9815, GlaxoSmithKline) alone or adjuvanted by 21 μg of PapMV nanoparticles. Bleedings were performed at day 0 and 28 and bronchoalveolar lavages (BAL), at day 28 post-immunization.

### Antibody response quantification

BALs were performed using 700 μl of phosphate buffered saline and cells were purged from the samples by centrifugation. Blood sample were collected and centrifuged in BD Microtainer blood collection tubes (BD, Mississauga, Ontario, Canada) for 2 minutes at 10 000 × g. The harvested serums and BAL fluids were assayed for total IgG, IgG2a serotype and IgA against TIV or IgG2a against GST-NP by enzyme-linked immunosorbent assay (ELISA) as described elsewhere [7,13]. GST-NP is a C-terminal fusion of the influenza nucleoprotein to a glutathione S-transferase (GST) protein [7]. ELISAs were conducted by serial dilutions by two-fold steps starting at 1 in 50. Results are expressed as an antibody endpoint titer greater than threefold the background optical density values consisting of preimmune sera or control BAL fluids.

### Mice challenge with influenza virus

Mice were challenged 2 weeks after the last immunization with 250 plaque-forming units (pfu) of A/WSN/33(H1N1) influenza virus by 50 μl intranasal instillation. Weight losses and symptoms were monitored for 14 days post-infection. Symptoms are rated from 0 to 4 (no symptoms (0), lightly spiked fur and curved back [1], spiked fur and curved back [2], difficulty to move and light dehydration [3], severe dehydration, lack of reflex and ocular secretion [4]), where 4 is the highest score and mice are euthanized. Mice that lost more than 20% of their initial weight are also euthanized.

### Statistical analysis

Data from ELISA and challenge (day 8 weight losses and symptoms) were analyzed with a parametric ANOVA test. Tukey’s post tests were used to compare differences among groups of mice. Kaplan-Meier survival curves were analysed by the log rank test. Values of *p < 0.05, **p < 0.001 and ***p < 0.0001 were considered statistically significant. Statistical analyses were performed using GraphPad PRISM 5.01.

### Ethics statement

All the work with animals has been done with institution approved ethics protocol by the “Comité de Protection des Animaux - CHUQ (CPA-CHUQ)”. The approval of this project is found under the authorization number 2010–148.

## Results and discussion

**Figure 1:**
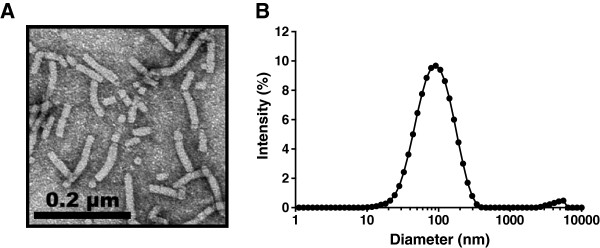
**Structure of the PapMV nanoparticles.** PapMV nanoparticles were observed with a transmission electron microscope and by dynamic light scattering. PapMV nanoparticles have a filamentous rod-shape structure **(A)** with an average length of 100 nm as shown by dynamic light scattering **(B)**.

PapMV nanoparticles are composed of the recombinant coat proteins of PapMV purified from *E. coli* and self-assembled around a single-stranded RNA. Transmission electron microscopy revealed that PapMV nanoparticles have a filamentous rod shape structure (Figure 1A) and an average length of 100 nm, as confirmed by dynamic light scattering (Figure 1B).

**Figure 2:**
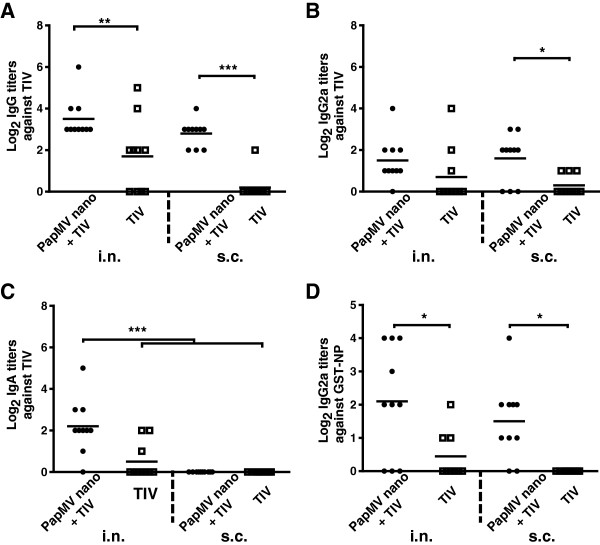
**PapMV nanoparticles improve the mucosal antibody response against TIV and NP in the lungs.** Balb/C mice (10/group) were vaccinated twice at a 14-day interval with TIV alone or adjuvanted by PapMV nanoparticles by intranasal (i.n.) or subcutaneous (s.c.) routes. Bronchoalveolar lavages were performed at day 28 and ELISA were conducted to evaluate the levels of total IgG **(A)**, IgG2a **(B)** or IgA **(C)** against TIV and IgG2a against NP **(D)**. Titers against TIV or GST-NP were not detected in the blood or BAL of PapMV nanoparticles or buffer vaccinated mice. *P < 0.05, **P < 0.01 and ***P < 0.001.

**Figure 3:**
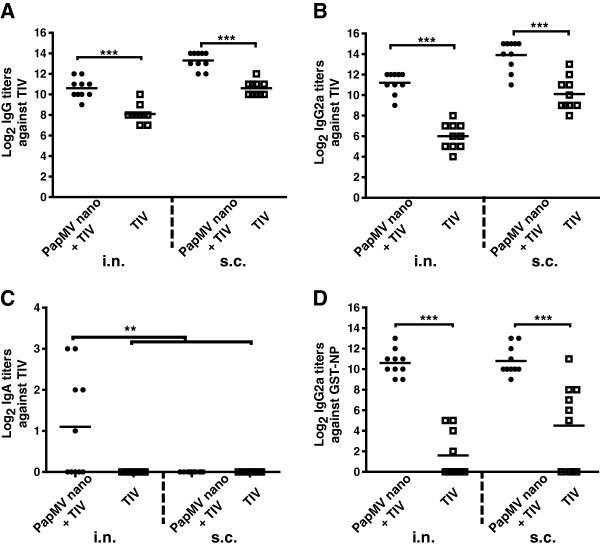
**PapMV nanoparticles improve the systemic antibody response against TIV and NP.** Balb/C mice (10/group) were vaccinated twice at a 14-day interval with TIV alone or adjuvanted with PapMV nanoparticles by intranasal (i.n.) or subcutaneous (s.c.) routes. Bleedings were performed at day 28 and ELISA were conducted to evaluate the levels of total IgG **(A)**, IgG2a **(B)** or IgA **(C)** against TIV and IgG2a against NP **(D)**. Titers against TIV or GST-NP were not detected in the blood or BAL of PapMV nanoparticles or buffer vaccinated mice. *P < 0.05, **P < 0.01 and ***P < 0.001.

To evaluate the potential of PapMV nanoparticles as a mucosal adjuvant, mice were immunized twice at 14 days interval by either the i.n. or s.c. routes with TIV alone or adjuvanted with PapMV nanoparticles. Antibodies levels against TIV in the BAL (Figure 2) and in the blood (Figure 3) were measured by ELISA at day 28. We showed that TIV adjuvanted with PapMV nanoparticles administered either by the i.n. or s.c. improved and potentiate the TIV as observed by the levels of total IgG titers directed against TIV (Figure 2A). IgG2a titers in the BAL showed significant higher levels in the aduvanted group only when administered by the s.c. route (Figure 2B). Interestingly, the group showing the highest IgA titers in the BAL was the adjuvanted TIV administered by the i.n. route (Figure 2C) showing clearly the effectiveness of PapMV nanoparticles to improve the mucosal antibody response in the lung. The ELISA also revealed that the adjuvanted vaccine broaden the immune response to the TIV antigens through a significant increased in the lungs of the IgG2a titers directed to the influenza NP (Figure 2D), a highly conserved protein through all the strains of influenza often used in experimental influenza vaccines [7,14,15]. NP has also been identified as a key antigen to trigger cross-protection to influenza viruses [8,16-18]. It is therefore a good strategy to increase the immune response directed to this antigen using PapMV nanoparticles to broaden and improve protection to this virus. Through the course of the experiment, we have not notice any sign of toxicity associated with the use of PapMV nanoparticles.

In the blood, the levels of total IgG or IgG2a were higher than in the BAL with either route of immunization (Figure 3). We also showed that the titers in total IgG or IgG2a directed to TIV or GST-NP were similar when the adjuvanted formulation was administered by the i.n. or the s.c. route (Figure 3A-B, D). As expected, only the adjuvanted vaccine administered by the i.n. route could trigger in the blood a significant amount of IgA directed to TIV (Figure 3C). Titers against TIV or GST-NP were not detected in the blood or BAL of PapMV nanoparticles or buffer vaccinated mice.

**Figure 4:**
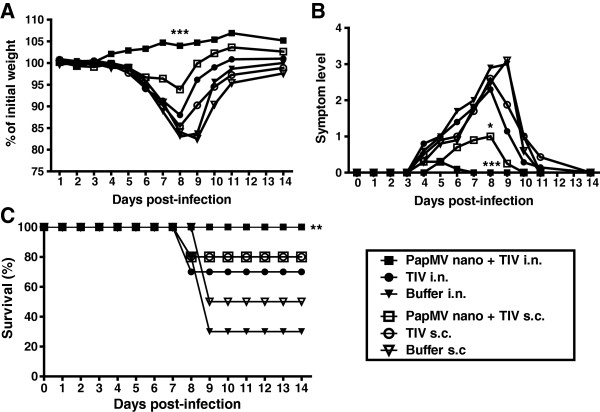
**Mice vaccinated with PapMV nanoparticles adjuvanted TIV showed an increased protection to an influenza challenge with influenza strain normally lethal to TIV vaccinated mice.** Mice vaccinated twice with TIV adjuvanted or not by PapMV nanoparticles by intranasal (i.n.) or subcutaneous (s.c.) routes were challenged with 250 pfu of A/WSN/1933 (H1N1) influenza virus. Mice were monitored for weight loss **(A)**, symptoms levels **(B)** and survival **(C)** for 14 days. Statistical analysis is applied between groups of the same immunization ways. *P < 0.05, **P < 0.01 and ***P < 0.001.

We previously showed that mice immunized s.c. with the TIV adjuvanted with PapMV nanoparticles induced protection against an strain of influenza that overcome the protection induced by the TIV [8]. In this study, we wish to validate that the adjuvanted TIV with PapMV nanoparticles administered by the intranasal route can also elicit this kind of protection. Therefore, we challenged vaccinated mice with A/WSN/33(H1N1), a strain of influenza that was previously showed to overcome the protection induced by the TIV. As expected, mice vaccinated with TIV by either route of immunization were not protected and showed major weight losses (more than 15%) (Figure 4A) and strong symptoms (Figure 4B); while mice immunized by the i.n. route with the PapMV adjuvanted TIV showed a weight increased (Figure 4A) without any symptoms during infection (Figure 4B). Mice immunized with the same formulation by the s.c. route lost 7% of their initial weight and showed mild symptoms at the infection peak (day 8) (Figure 4A,B). Finally, only mice immunized by the i.n. route with the adjuvanted TIV formulation showed 100% survival (Figure 4C). The PapMV adjuvanted i.n. vaccine generated a robust protection to the infection by an influenza strain normally lethal to TIV vaccinated mice. PapMV nanoparticles is therefore an efficient mucosal adjuvant with strong potential for its used in human.

## Conclusions

In conclusion, we demonstrate for the first time that PapMV nanoparticle is an effective and potent mucosal adjuvant for vaccination against a respiratory disease. This technology fulfills an important medical need for a safe mucosal adjuvant that broadens the protection of the TIV.

## Abbreviations

PapMV: Papaya mosaic virus; TIV: Trivalent inactivated flu vaccines; HA: Hemagglutinin; BAL: Bronchoalveolar lavage; ELISA: Enzyme-linked immunosorbent assay; NP: Nucleoprotein; GST: Glutathione S-transferase; Pfu: Plaque-forming units; i.n: Intranasal; s.c: Subcutaneous.

## Competing interests

Author Denis Leclerc and Pierre Savard are shareholder of the company FOLIA BIOTECH INC., a start-up company that has the mandate to exploit commercially this technology to improve and design new vaccines. This does not alter the authors’ adherence to all the journal policies.

## Authors’ contributions

GR performed mice experiments and drafted the manuscript. CM performed mice experiments and immunoassays and helped to draft the manuscript. GR and CM are considered to have equally contributed to this article. AR performed mice experiments and helped to draft the manuscript. MB, MELG and PS engineered the nanoparticles. DL supervised the study and revised the manuscript. All authors read and approved the final manuscript.
